# Volume of the effect compartment in simulations of neuromuscular block

**DOI:** 10.1186/1742-4682-2-41

**Published:** 2005-10-03

**Authors:** Vladimir Nigrovic, Johannes H Proost, Anton Amann, Shashi B Bhatt

**Affiliations:** 1 Department of Anesthesiology, Medical University of Ohio, Toledo, OH, USA; 2 Research Group for Experimental Anesthesiology and Clinical Pharmacology, University Hospital Groningen, Groningen, The Netherlands; 3 Department of Anesthesiology and Critical Care Medicine, Leopold-Franzens University, Innsbruck, Austria, and Department of Environmental Sciences, The Swiss Federal Institute of Technology, Zürich, Switzerland

## Abstract

**Background:**

The study examines the role of the volume of the effect compartment in simulations of neuromuscular block (NMB) produced by nondepolarizing muscle relaxants.

**Methods:**

The molar amount of the postsynaptic receptors at the motor end plates in muscle was assumed constant; the apparent receptor concentration in the effect compartment is the ratio of this amount and the volume arbitrarily assigned to the effect compartment. The muscle relaxants were postulated to diffuse between the central and the effect compartment and to bind to the postsynaptic receptors. NMB was calculated from the free concentration of the muscle relaxant in the effect compartment.

**Results:**

The simulations suggest that the time profiles of NMB and the derived pharmacokinetic and pharmacodynamic variables are dependent on the apparent receptor concentration in the effect compartment. For small, but not for large, volumes, times to peak submaximal NMB are projected to depend on the magnitude of NMB and on the binding affinities.

**Conclusion:**

An experimental design to estimate the volume of the effect compartment is suggested.

## Background

In the majority of the pharmacokinetic-pharmacodynamic (PK-PD) models proposed to simulate neuromuscular block (NMB) [1-3], the volume of the effect compartment is postulated to be negligibly small or the compartment is postulated to contain a negligibly small amount of the muscle relaxant. The models simulate NMB based on the concentration of the muscle relaxant in this compartment using the equation of Hill. Binding of muscle relaxants to the postsynaptic receptors at the motor end plates is not considered. Because muscle relaxants produce NMB by binding to these receptors, consideration of the interaction of muscle relaxants with the receptors represents a more realistic approach and an advancement in simulations [4-6]. Donati and Meistelman [5] were the first to consider binding of muscle relaxants to the receptors. These investigators suggested that the receptor concentration in the effect compartment is 2.8·10^-7 ^M, but the volume of the effect compartment was assumed to be negligibly small. Given a fixed amount of postsynaptic receptors, a finite receptor concentration is not compatible with a negligibly small volume of the effect compartment.

We decided to examine the role of the volume of the effect compartment in a pharmacokinetic-pharmacodynamic model for NMB and were interested in answering the following questions: (1) Is it necessary to postulate a negligibly small amount of a muscle relaxant in the effect compartment? (2) Do the projections from simulations using a small or a large volume of the effect compartment differ? If so, what are the differences? (3) Can the simulations suggest an experimental design suitable to test whether the volume of the effect compartment is negligibly small or a large volume may be more appropriate?

## Methods

### General approach

(1) The amount of the postsynaptic receptors at the motor end plates in muscle, in terms of mol per kg body weight, was assumed constant and the receptors uniformly diluted in the effect compartment. (2) The plasma concentrations of a hypothetical muscle relaxant after administration of an intravenous bolus dose, defined by an arbitrary multiexponential equation, are labeled target concentrations. In the simulations, the target plasma concentrations fulfill the role of the experimentally determined plasma concentrations. (3) A PK-PD model was designed *a priori *to include an effect compartment of an assigned volume. The pharmacokinetic parameters in the model were defined by the postulate that the concentrations in the central compartment (compartment1) fit the target plasma concentrations. (4) The muscle relaxant diffuses from the central to the effect compartment. (5) Pharmacodynamic parameters were obtained from the postulate that peak neuromuscular block from a bolus ED50 dose occurs at 4.5 minutes after injection. The peak concentration of the muscle relaxant in the effect compartment at this moment corresponds to the IC50 concentration. (6) The relationship between NMB and the free concentrations of the muscle relaxant in the effect compartment is defined by the Hill equation.

### The target plasma concentrations

Muscle relaxant D was postulated to display linear pharmacokinetics. The triexponential equation that defines the time course of the molar amounts of the muscle relaxant in plasma is given by (braces indicate molar amounts):



Here, N, O, and P (P = 1 - N - O) are fractions of the dose that are eliminated from plasma with the first order rate constants *λ*_N_, *λ*_O_, and *λ*_P_, respectively. The dose is in mol·kg^-1^. Division of the equation by V_C_, V_C _expressed in L·kg^-1^, converts the amounts in plasma to molar concentrations. V_C _represents the volume of the space into which the muscle relaxant is uniformly diluted at time t = 0, *i.e*., at the moment of bolus intravenous injection.

The values assigned to the parameters in the triexponential equation were based on the following postulates: For the hypothetical muscle relaxant D, V_C _approximates the volume of plasma and V_SS_, the volume of distribution at steady state, approximates the volume of the extracellular space. The dose that produces NMB50, *i.e*., ED50, is defined by the postulate that the concentration in plasma at 4.5 min after bolus intravenous injection is [D]_plasma _= IC50. The definition of IC50 is provided below. The following values satisfy these requirements:

N = 0.71; O = 0.192; P = 0.098

*λ*_N _= 1.3 min^-1^; *λ*_O _= 0.31 min^-1^; *λ*_P _= 0.0231 min^-1^

V_C _= 0.044 L·kg^-1 ^V_SS _= 0.28 L·kg^-1^

Compartmental interpretation of the triexponential decay of the plasma concentrations yields the following parameters for the standard 3-compartment pharmacokinetic model assuming a mammillary arrangement of the compartments and elimination only from compartment_1_[7]:

V_1 _= V_C _= 0.044 L·kg^-1 ^k_10 _= 0.1848 min^-1^

k_12 _= 0.3771 min^-1 ^k_21 _= 0.5581 min^-1^

k_13 _= 0.4229 min^-1 ^k_31 _= 0.0902 min^-1^

### Estimation of the receptor amount

The molar amount of receptors per kg body weight was estimated based on the following assumptions: One hundred g of muscle is represented as a cube with side length of 4.64 cm, *i.e*., specific density of muscle ~ 1. There is 430 g muscle per kg body weight. The muscle fibers are densely packed cylinders with the diameter of 50 *μ*m and the length of 4.64 cm (928 rows × 928 columns of fibers in a cross section perpendicular to the length of the fibers). Each muscle fiber has one motor end plate with 2.1·10^7 ^receptors at each end plate [8,9].

### The PK-PD Model

The pharmacokinetic model consists of four compartments: the central (compartment_1_), two peripheral (compartment_2 _and compartment_3_), and the effect compartment in mammillary arrangement with elimination from the central compartment. The model is defined in terms of the amounts of the muscle relaxant present in each compartment and the amount eliminated from the body. Transport between the central and the effect compartment is defined as diffusion according to the concentration gradient of the free muscle relaxant in both compartments. As a result, at the moment when the free muscle relaxant attains the peak concentration in the effect compartment and there is no net transport between the compartments (steady state), the concentrations in the two compartments are equal. In the model, this constraint necessitates that the transport rate constant into the effect compartment, k_1*e*_, be defined in terms of the transport rate constant from the effect to the central compartment, k_e1_. Hence, k_1*e *_= (V_e_/V_1_)·k_e1_, where V_e _and V_1 _represent the volumes of the effect and the central compartments, respectively. The volume of the central compartment is known (V_1 _≈ V_C _in the triexponential function). The volume of the effect compartment was assigned different values. Hence, the amounts of D in the central and the effect compartments may be converted to concentrations. Compartment_2 _and compartment_3 _are defined only in terms of the amounts present in them.

The amount of receptors in the effect compartment is constant and independent of the volume assigned to the effect compartment. A small assigned volume results in a high receptor concentration, while the concentration is low in the large effect compartment.

For an assigned volume of the effect compartment (V_e_), the pharmacokinetic parameters in the PK-PD model were estimated in a two-step procedure. In the first step, the parameter k_e1 _was obtained using the following constraints: dose = ED50, the amounts in plasma as defined by the triexponential equation, and the maximal NMB = 50% attained at 4.5 min after administration of the muscle relaxant. In the second step, the parameters V_1_, k_10_, k_12_, k_21_, k_13_, and k_31 _were estimated using the following constraints: dose = ED50 and k_e1 _fixed to the value obtained in the first step. The parameters were fitted by minimizing the sum of squared differences between the logarithms for the calculated concentrations in compartment_1 _and the target concentrations in plasma. The evaluations were carried out at 250 time points from t = 0 to t = 25 min and at 50 points for t = 25 to t = 50 min after administration. Goodness-of fit was expressed as the coefficient of variation (CV in % ^1^) of the differences between the two time profiles.

Interaction between the muscle relaxant and the postsynaptic receptors was defined in terms of the association, k_assoc_, and dissociation, k_dis_, rate constants. We assumed that each receptor possesses only a single binding site for the muscle relaxant. The ratio k_dis_/k_assoc _defines the equilibrium dissociation constant, K_D_. The inverse of K_D _defines the affinity of the receptors for the muscle relaxant.

The values of all the mentioned parameters are listed in Table 1. The set of five ordinary differential equations defining the amounts of the muscle relaxant in the four compartments and the amount of the complex with the receptors in the effect compartment is presented in the Appendix.

**Table 1:** Pharmacokinetic and pharmacodynamic parameters for the PK-PD model. The volume of the effect compartment (V_e_) was postulated to be either small (SMALL) or large (LARGE).

**Parameter**	**Unit**	**SMALL**		**LARGE**
ED50	mol·kg^-1^		2.2325·10^-7^	
V_1_	L·kg^-1^	0.0440		0.0434
k_10_	min^-1^	0.1847		0.1795
k_12_	min^-1^	0.3769		0.3574
k_21_	min^-1^	0.5587		0.7663
k_13_	min^-1^	0.4226		0.1981
k_31_	min^-1^	0.0909		0.0581
k_assoc_	M^-1^·min^-1^		2.4·10^10^	
{R}_total_	mol·kg^-1^		1.2921·10^-10^	
				
V_e_	L·kg^-1^	4.4·10^-5^		9.23·10^-2^
k_e1_	min^-1^	0.6159		0.1477
[R]_total_	M	2.9367·10^-6^		1.4·10^-9^
Onset time	min		4.50	

### Calculation of NMB

The intravenous bolus dose of the muscle relaxant required to produce a half-maximal NMB, NMB50, is labeled ED50. We postulated that NMB50 is attained at 4.5 min after the bolus injection. The peak concentration of the free muscle relaxant in the effect compartment established by ED50 is IC50. At 4.5 min after injection, [D]_plasma _= peak [D]_e _= IC50. The fractional receptor occupancy by the muscle relaxant (Occ) at NMB50 is labeled Occ_NMB50 _and assigned a value of 0.875 [10]. Because K_D _= [D]_e_·(1 - Occ)/Occ, and at NMB50 [D]_e _= IC50 and Occ = Occ_NMB50 _= 0.875, it follows that IC50 = 7·K_D_.

Neuromuscular block (NMB) was calculated using the Hill equation, the free concentrations of the muscle relaxant in the effect compartment, [D]_e_, and two parameters: *γ *and IC50 (*γ *= 4 and IC50 = 7·K_D_, Eq 1 in Appendix).

To describe quantitatively the simulated NMB as a function of doses used to establish the peak concentrations in the effect compartment, the values for NMB calculated from peak [D]_e _were plotted as a function of doses of the muscle relaxant. A modified equation of Hill (Eq. 2, Appendix) was fitted to these points using the program TableCurve2D from SPSS, Chicago, IL, and the fitted estimates of the exponent *γ*_f _and ED50_f _are reported.

All calculations were performed independently using the programs MATHEMATICA (version 5.1) from Wolfram Research, Inc., Champaign, IL, MULTIFIT and PKPDFIT written by J.H. Proost, and MATLAB (version 6.1.0.450(R12.1)) from The Mathworks Inc., Natick, MA.

## Results

The estimated total molar amount of receptors at the motor end plates in muscles is {R}_total _= 1.2921·10^-10 ^mol·kg^-1^. Receptor concentration in the effect compartment is the ratio of this amount and the volume assigned to the effect compartment.

### Simulations with a small or a large volume assigned to the effect compartment

**Figure 1:**
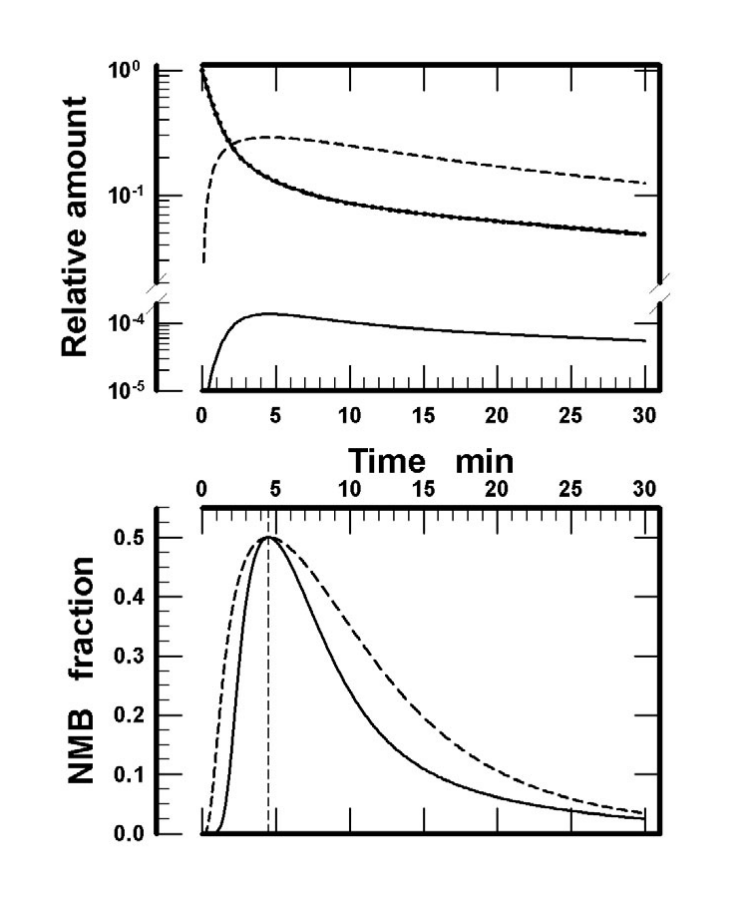
*Upper panel: *The amounts of muscle relaxant D (K_D _= 1·10^-7 ^M) in plasma and compartment_1 _and the free amounts in the effect compartment assigned a small (V_e _= 4.4·10^-5 ^L·kg^-1^) or a large (V_e _= 9.23·10^-2 ^L·kg^-1^) volume. All the amounts are normalized to the injected dose (= ED50 = 2.23·10^-7 ^mol·kg^-1^). Solid and dashed lines indicate the amounts contained in the small and the large effect compartment, respectively. Filled circles denote the target amounts in plasma defined by the triexponential function. The three curves for the amounts in plasma overlap. The estimates were obtained at 0.1 min intervals. *Lower panel: *Time course of the neuromuscular block (NMB) by ED50 of the muscle relaxant D. NMB was calculated using Eq 1 (Appendix), [D]_e _for the small and large volume of the effect compartment presented in the upper panel, and by setting *γ *= 4.0 and IC50 = 7·10^-7 ^M. The lines are identical to those in the upper panel for the small and large volume of the effect compartment.

For the initial simulations, V_e _was assigned the value of 0.001·V_C_, *i.e*., V_e _= 4.4·10^-5 ^L·kg^-1 ^[11], for the small and 0.0923 L·kg^-1 ^for the large effect compartment. The latter approximates the volume of the interstitial space in muscle. Receptor concentrations in the effect compartment were: [R]_total _= 2.94·10^-6 ^M and 1.4·10^-9 ^M for the small and large volume, respectively. The hypothetical muscle relaxant D was assigned K_D _= 1·10^-7 ^M. The assignment defined ED50 as ED50 = 2.23·10^-7 ^mol·kg^-1^. Optimal estimates of the pharmacokinetic parameters, including k_e1_, were obtained as described in the Methods section. The target amounts of the muscle relaxant in plasma and those estimated in compartment_1 _as well as the amounts in the small and the large effect compartments are graphically presented in the upper panel of Figure 1. The three curves for the amounts in plasma overlap. The good fit of the amounts in compartment_1 _to the target amounts in plasma is evident from the small values of the coefficient of variation, 0.0007% for the small and 0.7% for the large volume of the effect compartment. The peak free amount of D in the small effect compartment constitutes a small fraction of ED50, 1.38·10^-4^. On the other hand, the peak free amount of D in the large effect compartment accounts for a sizable fraction of ED50, 0.289 (upper panel in Figure 1). The PK-PD model that includes a large effect compartment requires intercompartmental transport rate constants different from those for the small volume of the effect compartment (Table 1). The peak receptor occupancy, Occ = Occ_NMB50 _= 0.875, and the peak [D]_e _= IC50 = 7·K_D_, were attained at 4.50 min for either volume of the effect compartment. Hence, for both volumes the simulated peak NMB = NMB50 and occurs at 4.5 min after injection, but the time course of NMB is different between the small and large volumes of the effect compartment (lower panel in Figure 1). To reach the respective peaks at 4.50 min after the injection required k_e1 _that was approximately four times higher for the small than for the large effect compartment (Table 1).

The calculations were verified by calculating the sum of the amounts in the four compartments plus the amount eliminated from the body. For all times between 0 and 50 min after injection, the sum was equal to ED50. Expressed as fractions of the administered dose (= ED50), the peak amounts in compartment_2 _and compartment_3 _and the times after injection when the peaks were attained are for the small volume of the effect compartment 0.199 at 1.6 min and 0.483 at 7.3 min, respectively. For the large volume, the corresponding values are 0.158 at 1.3 min and 0.268 at 11.9 min.

Two additional observations were made during these simulations. First, exclusion of the small effect compartment from the PK-PD model only minimally influences the fit of the amounts in compartment_1 _to the target plasma amounts. The result is not unexpected, because the intercompartmental transport rate constants (microconstants) in the model with a small volume of the effect compartment (Table 1) are close to those in the standard 3-compartment model. Second, when the effect compartment in the PK-PD model was postulated not to contain the receptors, *i.e*., {R}_total _= 0, identical values of k_e1 _establish the peak free amount of D in the respective effect compartment at identical times (data not presented).

**Figure 2:**
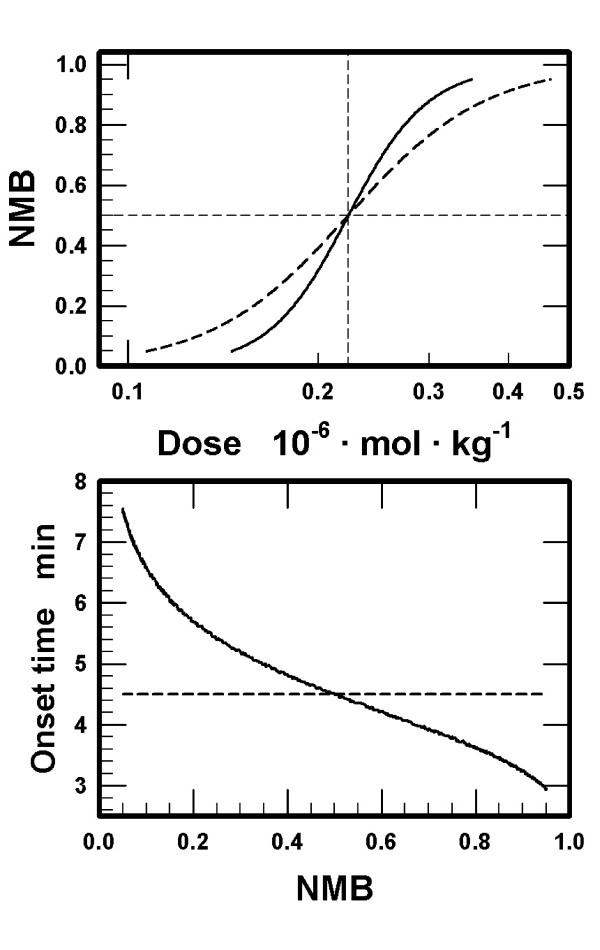
*Upper panel: *Neuromuscular block (NMB) calculated as a function of the peak concentrations of muscle relaxant D in the effect compartment using Eq 1 presented in the Appendix (*γ *= 4.0 and IC50 = 7·10^-7 ^M). The doses presented along the abscissa refer to the doses that established the peak concentrations. The range of NMB is from NMB05 to NMB95. One thousand logarithmically equidistant values were used for a 10-fold increase in doses. The volumes of the effect compartment and the lines are identical to those presented in Figure 1. *Lower panel: *Onset times as a function of the magnitude of NMB. Onset times are defined as the times after the bolus intravenous injection of muscle relaxant D needed to establish peak NMB, from NMB05 to NMB95. Other details are identical to those in the upper panel.

Based on the derived pharmacokinetic rate constants, NMB was simulated with different doses of D. One thousand points were selected for a 10-fold increase in doses. NMB was calculated with the peak free concentrations of D in the effect compartment using Eq 1 in the Appendix (*γ *= 4 and IC50 = 7·10^-7 ^M). The relationship between NMB and the doses that produced the peak concentrations differed between the models (upper panel in Figure 2 for NMB = 0.05 to NMB = 0.95, *i.e*., NMB05 to NMB95). To obtain a quantitative estimate for the difference, equation of Hill (Eq 2) was fitted to both sets of points to describe the relationship between NMB and the injected doses. The fit was excellent for both sets (r^2 ^> 0.9999, the number of points, n, = 381 for the small and n = 641 for the large volume of the effect compartment). The 95% confidence interval (95%CI) for the fitted *γ*_f _was 6.819 to 6.838 for the small and 4.0040 to 4.0041 for the large effect compartment. The 95%CI for the fitted ED50_f _were (2.235 to 2.236)·10^-7 ^mol·kg^-1 ^and (2.23256 to 2.23258)·10^-7 ^mol·kg^-1^, respectively.

The onset times for NMB05 to NMB95 differed between the models assigned different volumes of the effect compartment (lower panel in Figure 2). The model with the large effect compartment projected that the onset times were nearly independent of the magnitude of NMB. The model incorporating a small volume of the effect compartment projected an inverse relationship between the onset times and the magnitudes of NMB. For NMB < NMB50, the onset times were longer, and for NMB > NMB50 the onset times were shorter than those projected by the model with a large effect compartment.

### Simulations with different volumes assigned to the effect compartment using ED50

**Figure 3:**
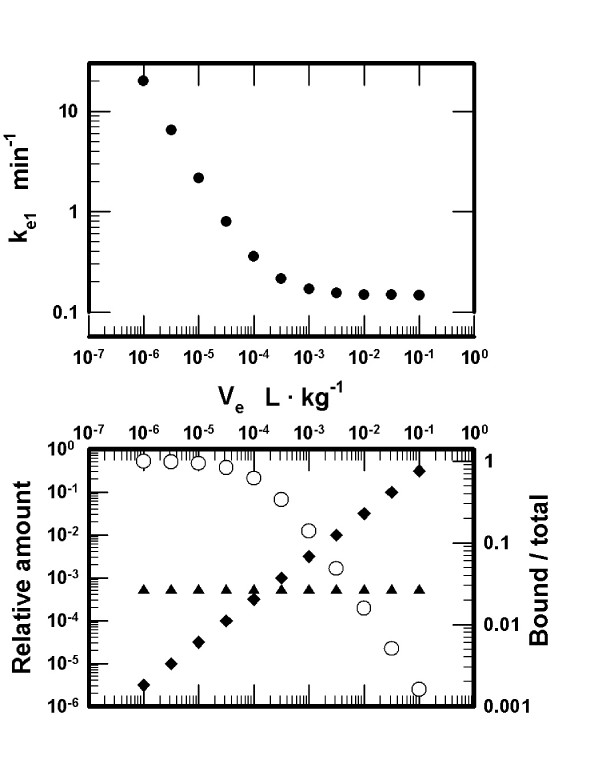
*Upper panel: *Values estimated for the transport rate constant between the effect and the central compartment, k_e1_, as a function of the volume assigned to the effect compartment, V_e_. The dose of the muscle relaxant D = ED50 = 2.23·10^-7 ^mol·kg^-1 ^and K_D _= 1·10^-7 ^M. *Lower panel: *Amounts of the muscle relaxant D (left Y-axis) bound to the receptors (filled upright triangles) and the amounts free (filled diamonds). The amounts are normalized to the injected dose presented in the upper panel. The ratio of the bound to the total amounts (empty circles; total = bound + free; right Y-axis) in the effect compartment is presented as a function of the volume assigned to the effect compartment.

Next, the influence of the volume assigned to the effect compartment was examined systematically. The volumes varied from 1·10^-6 ^to 1·10^-1 ^L·kg^-1 ^(11 logarithmically equidistant values). The pharmacokinetic parameters, including k_e1_, were estimated as previously stipulated, *i.e*., ED50 dose establishes peak receptor occupancy = Occ875 and peak [D]_e _= IC50 at 4.5 min after injection. The coefficient of variation for the fit of the concentrations of D in compartment_1 _to the target plasma concentrations was CV = 0.84% for the largest and = 0.0006% for the smallest volume. The values for k_e1 _as a function of the assigned volumes, estimated with ED50 and using the same muscle relaxant (K_D _= 1·10^-7 ^M), are presented in the upper panel of Figure 3. The results demonstrate that k_e1 _increases markedly for the smaller values of V_e_. The relative amounts of D bound to the receptors, the amounts free in the effect compartment, and the ratio of the bound to the total amount in the effect compartment with ED50 show (lower panel in Figure 3) that for all volumes the amounts of D bound to the receptors are constant. For smaller volumes the bound amounts make up nearly all of D present in the effect compartment, while for larger volumes the total amount of D is nearly completely accounted for by the free amount.

### Simulations using different volumes and different doses

**Figure 4:**
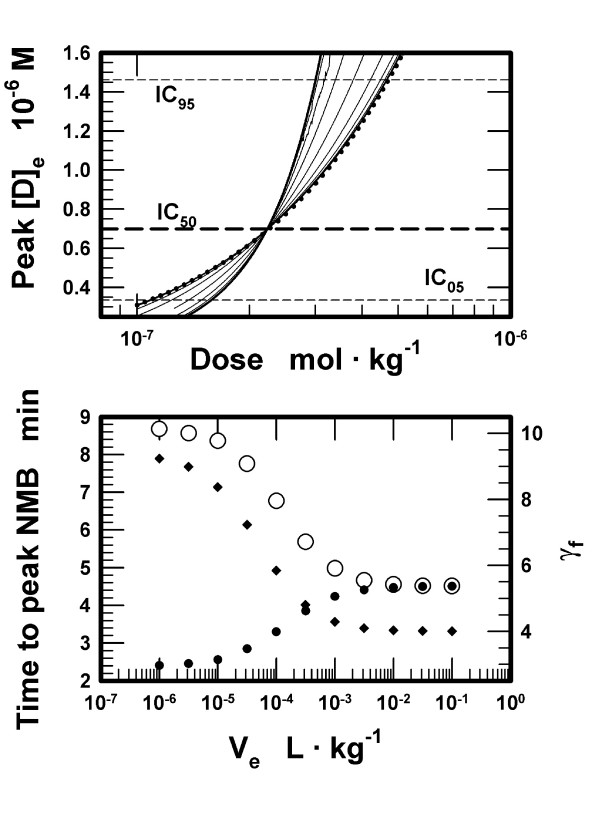
*Upper panel: *The peak free concentrations of muscle relaxant D in the effect compartment calculated with variable doses of D as a function of the volumes assigned to the effect compartment. The assigned volumes were: 1·10^-6^, 3.16·10^-6^, 1·10^-5^, 3.16·10^-5^, 1·10^-4^, 3.16·10^-4^, 1·10^-3^, 3.16·10^-3^, 1·10^-2^, 3.16·10^-2^, and 1·10^-1 ^L·kg^-1^. The bold solid and the dotted lines indicate the lowest and the highest assigned volumes, respectively. Concentrations for the intermediate volumes are indicated in sequence by thin solid lines. The three dashed lines parallel with the X-axis represent the free concentration of D for NMB95 (IC95, upper line), for NMB50 (IC50, middle line) and for NMB05 (IC05, lower line). The concentrations for IC05 and IC95 were calculated based on *γ *= 4.0 (Eq 1, Appendix). *Lower panel: *Times to NMB05 (open circles) and NMB95 (filled circles, left Y-axis) as a function of the volumes assigned to the effect compartment, V_e_. Neuromuscular block was calculated using Eq 1 (IC50 = 7·10^-7 ^M, *γ *= 4) and the peak free concentrations of D presented in the upper panel. The values of the exponent *γ*_f _(filled diamonds, right Y-axis) were obtained by fitting Eq 2 to the calculated NMB.

We used the set of pharmacokinetic parameters obtained for each assigned volume, but now varied the dose using 1000 values for a ten-fold increase. The results are presented in Figure 4. Increasing doses increase the peak free concentrations of D for each volume of the effect compartment (upper panel in Figure 4). The increase is steepest for the smallest volume and the slopes decrease for the larger assigned volumes. The estimated peak free concentrations of D in the effect compartment were used to calculate NMB (IC50 = 7·10^-7 ^M and *γ *= 4, Eq 1 in Appendix). The values of NMB from NMB05 to NMB95 as calculated using [D]_e _were plotted against the injected doses separately for each assigned volume, similarly to the results presented in the upper panel of Figure 2. The modified equation of Hill (Eq 2, Appendix) was fitted to each of these 11 sets of points to define NMB as a function of the injected doses. The fit was excellent (r^2 ^> 0.9996 for n between 314 to 641 points). The fitted values of *γ*_f _are presented in the lower panel in Figure 4. The values increase markedly for smaller volumes. The 95%CI for the eleven fitted estimates of ED50_f _varied between (2.225 to 2.226)·10^-7 ^mol·kg^-1 ^for the smallest and (2.25720 to 2.25722)·10^-7 ^mol·kg^-1 ^for the largest volume. These simulations permitted us to estimate the times to NMB05 and NMB95 (onset times). The results are presented in the lower panel of Figure 4. Onset times for NMB05 and NMB95 differ widely for the small volumes, but the differences progressively decrease for larger volumes of the effect compartment. The onset times for NMB05 and NMB95 are nearly identical for the largest assigned volume.

### Simulations with different binding affinities assigned to muscle relaxants

The PK-PD model was also tested with two additional muscle relaxants using the previously defined small and large volumes of the effect compartment. One muscle relaxant, D_2_, was assigned a 10 times lower affinity for the binding sites at the receptors, K_D2 _= 1·10^-6 ^M. The other, D_3_, was assigned a 10 times higher affinity, K_D3 _= 1·10^-8 ^M. The assignments changed the respective k_diss_, but not k_assoc_. Two series of simulations were performed. In the first series, all the pharmacokinetic constants, including k_e1_, were those defined previously for either the small or the large volume of the effect compartment and for the muscle relaxant with K_D _= 1·10^-7 ^M (Table 1). A 100-fold increase in affinities was projected to require 16.3 times lower ED50 for the small volume (ED50 = 1.144·10^-6 ^mol·kg^-1 ^for D_2 _and ED50 = 7.018·10^-8 ^mol·kg^-1 ^for D_3_), but a 98.9 times lower ED50 (ED50 = 2.230·10^-6 ^mol·kg^-1 ^for D_2 _and ED50 = 2.255·10^-8 ^mol·kg^-1 ^for D_3_) for the model with a large volume of the effect compartment. For the small effect compartment, the times to reach NMB50 were 1.82 min for D_2 _and 34.74 min for D_3_. In the model with a large effect compartment, the times to NMB50 differed only minimally, from 4.50 min for D_2 _to 4.54 min for D_3_.

In the second series of simulations, we postulated that ED50 of either D_2 _or D_3 _produces NMB50 at 4.5 min after injection using either the small or the large volume of the effect compartment. The doses producing NMB50 were related to the assigned K_D _values, *i.e*., ED50 = 2.2325·10^-6 ^mol·kg^-1 ^for D_2 _and ED50 = 2.2325·10^-8 ^mol·kg^-1 ^for D_3_. These doses establish plasma concentrations at 4.5 min [D]_plasma _= IC50 = 7·K_D _= peak [D]_e_. In the model containing a small volume of the effect compartment, the postulate was satisfied by k_e1 _= 0.196 min^-1 ^for D_2 _and k_e1 _= 4.710 min^-1 ^for D_3_. For the large volume, the estimates of k_e1 _were 0.1475 min^-1 ^for D_2 _and 0.1498 min^-1 ^for D_3_.

## Discussion

The simulations suggest that the volume of the effect compartment *per se *is not the critical parameter in a PK-PD model for nondepolarizing muscle relaxants. If the effect compartment is postulated to be void of the postsynaptic receptors, then the peak concentration of free muscle relaxant in this compartment is attained at identical times using identical transport rate constant k_e1 _for any volume of the effect compartment. These conclusions agree with those obtained from PK-PD models assuming a negligibly small volume of the effect compartment and not taking into account binding of a muscle relaxant to the postsynaptic receptors [1-3]. However, NMB is produced not by the free molecules of muscle relaxants in the effect compartment, but by the molecules bound to the postsynaptic receptors at the motor end plates. Therefore, consideration of binding of muscle relaxants to the postsynaptic receptors in the effect compartment is advantageous in PK-PD modeling. The present simulations confirm the conclusion from the reports [4-6] that the receptor concentration in the effect compartment is a critical parameter in PK-PD modeling. The predictions from our simulations assuming a low or a high receptor concentration differ with respect to (1) the onset times to the peak but submaximal neuromuscular block for a single muscle relaxant (lower panel in Figure 2), (2) the time course of NMB using ED50 (lower panel in Figure 1), (3) the shape of the NMB-*versus*-dose curves (upper panel in Figure 2), (4) the estimates of k_e1 _(Table 1 and upper panel in Figure 3), and (5) the estimates of ED50 and the onset times as a function of affinities assigned to the muscle relaxants for binding to the receptors (D_2 _and D_3_, Results).

In the aforementioned models [4-6], receptor concentration was an explicit model parameter. In contrast, the present model defines the receptor concentration as the ratio between the constant amount of postsynaptic receptors and the variable volume assigned to the effect compartment. This approach allows PK-PD modeling without the constraint of a negligibly small effect compartment. The earlier models taking into account receptor concentration [4-6] assumed a negligibly small volume of the effect compartment. Given a fixed amount of postsynaptic receptors, a finite receptor concentration is not compatible with the negligibly small volume of the effect compartment. This is an inherent weakness of such models.

A compartment is defined by Jacquez [12] as "an amount of a material that acts kinetically like a distinct, homogenous amount". This is the reason that the five equations defining the transport and the distribution of a muscle relaxant in the body (Appendix) were formulated in terms of amounts rather than concentrations. The total amount of a drug in the body is represented by two, three, or more such compartments. The necessity to invoke more than a single compartment arises from the physico-chemical properties of the drug in relation to those structures in the body that prevent drug's uniform dilution. Anatomical structures and/or physiologic processes represent these barriers. For muscle relaxants, small hydrophilic cations with MW < 1000 da, the principal barriers are the capillary wall and the cellular membranes. It seems plausible to postulate that muscle relaxants diffuse through the pores in the capillary wall into the surrounding interstitial spaces. Diffusion across the cellular membranes is very unlikely due to the high hydrophilicity of the molecules. Therefore and as a first approximation, muscle relaxants remain diluted in a space limited to plasma and the interstitial space. The pharmacokinetic compartments for muscle relaxants likely represent the amounts of muscle relaxants in plasma and the interstitial spaces of different tissues.

In the muscle, muscle relaxants diffuse throughout the interstitial space, including the synaptic clefts at the motor end plates. There are no anatomical barriers between the interstitial space in muscle and the synaptic clefts to prevent diffusion of muscle relaxants into the synaptic clefts [13]. These considerations qualify the interstitial space in muscle, including the synaptic spaces, as a single pharmacokinetic compartment. The volume of the interstitial space in muscle defines the volume of this compartment.

Due to the presence of the postsynaptic receptors in the synaptic clefts, the

compartment represents the effect compartment for muscle relaxants. The functional receptors are immobile and are located exclusively within the synaptic clefts. Hence, interaction between the receptors and the free molecules of a muscle relaxant occurs due to diffusion of the free molecules of the muscle relaxant to the receptors. In effect, interaction between the two partners may be represented as proceeding in a space common to both, *i.e*., the interstitial space in muscle. Volume of this space defines the volume of the effect compartment, V_e_. We suggest that the apparent or the effective concentration of the postsynaptic receptors for the interaction with muscle relaxants is the ratio of the amount of receptors and the volume of interaction, [R]_total _= {R}_total_/V_e_.

Transport of a drug between two compartments is represented in a standard pharmacokinetic model by two first-order rate constants. A modification of this approach is needed, if the transport is assumed to proceed via diffusion. Occurrence of a peak amount in a non-central compartment suggests that at that moment there is no net transport. The postulate of transport via diffusion implies that the concentration of a muscle relaxant in the central and the peak concentration in the effect compartment are identical at that moment. In the simulations, the transport rate constant out of the effect compartment into compartment_1 _is represented by the symbol k_e1_. The rate constant in the opposite direction, k_1*e*_, is expressed as a function of k_e1_, *viz*., k_1*e *_= k_e1_·(V_e_/V_1_). The expression results from the postulate that the transport occurs via diffusion. We suggest that k_e1 _may be interpreted as the ratio of the plasma flow to the muscle and the volume of the interstitial space in muscle. For the adductor pollicis muscle and assuming plasma flow to the forearm or the hand of 0.9 to 4.7 mL·min^-1^·(100 g muscle)^-1 ^[14] and the volume of the interstitial space in muscle of 15 to 22 mL·(100 g muscle)^-1^, the value of the transport rate constant k_e1 _may be estimated to between 0.041 and 0.313 min^-1^.

The postulate seems plausible that the molar amount of the postsynaptic receptors is a physiologic constant. The value of the constant may be 10 times lower or 10 times higher than the assigned value (Table 1) without markedly altering the results of the simulations. The postulate of a constant amount of postsynaptic receptors permits the definition of the apparent receptor concentration in the effect compartment via the relationship [R]_total _= {R}_total_/V_e_.

The results of the simulations demonstrate that a PK-PD model may be constructed for a wide range of volumes assigned to the effect compartment. We examined V_e _from 1·10^-6 ^to 1·10^-1 ^L·kg^-1^and the corresponding apparent concentrations of the receptors. In general, smaller volumes require higher values of k_e1 _(upper panel in Figure 3), are associated with smaller total amounts of the muscle relaxant in the effect compartment and larger fractions of the muscle relaxant in the bound form (lower panel in Figure 3). The smaller volumes are compatible with the intercompartmental transport rate constants close to those in the standard 3-compartment pharmacokinetic model. For volumes < 1·10^-3 ^L·kg^-1^, the onset times of submaximal NMB are negatively correlated with the magnitude of NMB (lower panels in Figures 2 and 4). The onset times are also markedly dependent on the values assigned to the equilibrium dissociation constants for binding of the muscle relaxants to the receptors (muscle relaxants D_2 _and D_3_), higher affinities associated with prolonged onset times. All these findings change for V_e _> 1·10^-3 ^L·kg^-1 ^and the receptors concentrations < 1·10^-7 ^M (Figures 3 and 4). Specifically, the values of the rate constant k_e1 _become smaller and relatively independent of the assigned volumes (upper panel in Figure 3), the differences between onset times for NMB05 and NMB95 progressively disappear (lower panel in Figure 4), the affinities do not influence the onset to NMB50, and ED50 doses are proportional to K_D _(muscle relaxants D_2 _and D_3_, Results). It appears as if the value of V_e _of about 1·10^-3 ^L·kg^-1 ^and the receptor concentration ~ 1·10^-7 ^M represent the critical threshold for the difference between a "small" and a "large" volume of the effect compartment.

The results of the simulations reveal a difference in the slopes of the NMB curves when evaluated as a function of the injected doses of a muscle relaxant. As in the available pharmacodynamic models, NMB in the proposed model was calculated using the peak free concentration of a muscle relaxant in the effect compartment and two constants: *γ *and IC50 (Eq 1, Appendix). When the NMB, calculated using peak [D]_e_, was plotted as a function of the doses that produced these peak concentrations in the effect compartment, the fitted value of *γ*_f _(Eq 2, Appendix) was larger than *γ *used in the calculation of NMB from [D]_e _(upper panel in Figure 2 and lower panel in Figure 4) and the fitted values of *γ*_f _increase progressively for smaller volumes assigned to the effect compartment (lower panel in Figure 4). For volumes > 10^-3 ^L·kg^-1^, the fitted values of *γ*_f _approach the value of *γ *used in the calculations of NMB from [D]_e _(lower panel, Figure 4). The difference is due to the relationship between the peak concentrations of the free muscle relaxant in the effect compartment and the injected doses (upper panel in Figure 4). For volumes < 10^-3 ^L·kg^-1^, the peak concentrations increase rapidly with increasing doses. The steeper slope implies that the difference in doses producing IC05 and IC95, corresponding to NMB05 and NMB95, respectively, is smaller the smaller the volume assigned to the effect compartment. The narrower spread of these doses leads, in turn, to higher fitted values of *γ*_f _when NMB is represented as a function of the injected dose. To summarize, if V_e _< 10^-3 ^L·kg^-1^, then a correlation of NMB to the doses needed to establish the peak concentrations requires fitted values for *γ*_f _higher than the value of *γ *used in calculating NMB from [D]_e_. For V_e _> 10^-3 ^L·kg^-1^, the estimates of the fitted *γ*_f _approach the value of *γ *used in calculating NMB as a function of [D]_e_. Therefore, a comparison of *γ*, estimated in a PK-PD model and based on [D]_e_, with *γ*_f_, obtained experimentally in a NMB-*versus*-dose study, provides information about the volume of the effect compartment and the receptor concentration in it. The fitted values of ED50_f _are rather independent of the volumes assigned to the effect compartment and the estimates are close to the *a priori *defined ED50 used in calculating the target plasma concentrations.

The PK-PD models are based on two sets of experimental data: the time course of the plasma concentration of a muscle relaxant and the time course of NMB. The models simulate, and are applicable only to, the concentrations in plasma and in the postulated effect compartment. The amounts or concentrations in the other compartments and the amount eliminated from the body are not verifiable from the available experimental data. These compartments are included in the current PK-PD model solely to preserve mass balance and to fit the amounts or concentrations of D in compartment_1 _to the target plasma amounts or concentrations. *A posteriori *addition of a large effect compartment to the standard 3-compartment PK model alters the simulated amounts or concentrations in compartment_1 _and the fit of the standard 3-compartment model to the target plasma concentrations is lost. Realization of this fact was the primary reason for the postulate of a negligibly small effect compartment in the previously introduced PK-PD model [2]. However, as demonstrated in the current simulations, a PK-PD model may include an effect compartment of any volume and contain a sizable fraction of the injected dose, if the model is designed *a priori *and the pharmacokinetic rate constants, including k_e1_, adjusted so that the amounts in compartment_1 _represent as closely as possible the observed amounts in plasma. The fitting process is similar to that for fitting a standard pharmacokinetic model to the observed plasma concentrations. Alternatively and without prejudging mass transport from plasma to any compartment, the amounts in plasma may be described using a multiexponential equation without detriment to the pharmacodynamic part of the model.

## Conclusion

The simulations do not indicate whether a PK-PD model containing a small or a large effect compartment is more appropriate. The selection should be based on the results of prospective clinical experiments. The simulations suggest an optimal experimental design. The study needs to be conducted with several muscle relaxants. Several doses of each are selected to produce less than complete NMB, *e.g*., NMB10 to NMB90. The experiment needs to answer the following question: Is the onset time of submaximal NMB produced by a single muscle relaxant a function of the level of NMB? If the results with a single muscle relaxant show an inverse relationship between the level of NMB and the onset times, then the model containing a small volume of the effect compartment and a high receptor concentration is more appropriate. If the onset times are independent of the magnitude of the submaximal NMB, then the PK-PD model containing a large volume of the effect compartment and a low receptor concentration is more appropriate.

## Appendix

The pharmacokinetic part of the model was formulated with the volume of the effect compartment, V_e_, explicitly incorporated in the model. The following symbols are used: D for the muscle relaxant and R for the receptors. The braces denote molar amounts per kg body weight. The first and second subscript appended to the rate constants denote the number of the source and the target compartments, respectively. Subscript *e *denotes the effect compartment, *e.g*., k_1*e *_denotes the rate constant for the transport from compartment_1 _to the effect compartment and k_e1 _the transport in the reverse direction. The symbol {D}_e _denotes the free amount of D in the effect compartment.



DR represents the 1 : 1 complex of D with the receptors within the effect compartment. The differential equation for {DR} was derived from the differential equation for [DR] written in terms of the molar concentrations [D]_e_, [R]_total_, and [DR]. Multiplication of this equation by V_e _converts the concentrations into amounts. The definition of k_1*e *_in terms of _ke1_, *viz*., k_1*e *_= k_e1_·(V_e_/V_1_), results from the postulate of diffusion as the transport mechanism and implies that the peak concentration of D in the effect compartment equals the concentration in compartment_1 _at the same moment. The initial conditions at t = 0 are: {D}_1 _= dose, and {D}_2 _= {D}_3 _= {D}_e _= {DR} = 0.

The Hill equation for the calculation of NMB from the free molar concentrations of the muscle relaxant D in the effect compartment, [D]_e_, is given by:



where [D]_e _= peak {D}_e_/V_e _and IC50 = 7·K_D _= peak [D]_e _when Occ = 0.875. The exponent *γ *was arbitrarily assigned a value of 4.0.

A different form of the Hill equation was used to fit the calculated NMB (Eq.1) as a function of the doses producing the peak [D]_e_. The modified equation relates NMB to the injected doses:



The values for the exponent *γ*_f _and ED50_f _were derived in the fitting process.

## Competing interests

The author(s) declare that they have no competing interests.

## Authors' contributions

All authors have contributed equally to the conception and the design of simulations, to acquisition of data and data analysis, and to the preparation of the manuscript. All authors read and approved the final manuscript.

## Note

^1^, where [D_plasma_]_i _is the molar concentration of D in plasma calculated from the triexponential equation at time t_i_, [D_1_]_i _is the molar concentration in compartment_1 _at the same time, and n is the number of time points (= 300).
